# Effects of Barium Excess on the Performance of Multilayer Microtubular Proton Ceramic Electrochemical Hydrogen Pumps with Ba_x_Ce_0.7_Zr_0.1_Y_0.1_Yb_0.1_O_3−δ_ (x = 1.05, 1.10, 1.15)

**DOI:** 10.3390/membranes16090307

**Published:** 2026-09-20

**Authors:** Shao Zhang, Lihui Wang, Congcong Li, Mingming Wang, Zhigang Wang, Xiaoyao Tan

**Affiliations:** 1 State Key Laboratory of Advanced Separation Membrane Materials, College of Chemical Engineering and Technology, Tiangong University, Tianjin 300387, China; 2120020043@tiangong.edu.cn (S.Z.);; 2 Hebei Industrial Technology Research Institute of Membranes, Cangzhou Institute of Tiangong University, Cangzhou 061000, China

**Keywords:** multilayer microtubular structure, electrochemical hydrogen pump, barium excess, perovskite electrolyte

## Abstract

A series of Ba-excess Ba_x_Ce_0.7_Zr_0.1_Y_0.1_Yb_0.1_O_3−δ_ (B_x_CZYYb, x = 1.05, 1.10, 1.15) was employed as electrolytes. Multilayer microtubular protonic ceramic electrochemical hydrogen pumps (PCEHPs) with a current collector/anode/electrolyte/cathode/current collector architecture were fabricated by a triple-layer one-step co-spinning and co-sintering method. Their hydrogen separation performance was systematically investigated over the temperature range of 200–400 °C. The results show that the hydrogen pump employing the B_1.05_CZYYb electrolyte delivers the optimal performance. At 250 °C and a feed H_2_ concentration of 30 vol%, the hydrogen permeation flux achieves 1.01 mL min^−1^ cm^−2^, with Faradaic efficiency maintained above 95%. EDS line-scan results reveal the presence of Ni-rich precipitates on grain surfaces in the co-sintered electrolyte layer. It is speculated that, under the co-sintering conditions, excessive Ba may induce lattice distortion and reduce Ni solubility in the perovskite lattice, thereby promoting Ni exsolution at grain surfaces; the precipitated Ni could, in turn, hinder proton conduction and increase the ohmic resistance. These findings suggest that the hydrogen-permeation performance of multilayer PCEHPs is governed by the combined effect of Ba excess and Ni rather than by Ba doping alone. This work provides a valid experimental basis and theoretical reference for component optimization and structural design of high-performance co-sintered microtubular PCEHP devices.

## 1. Introduction

With the global energy structure shifting toward low-carbon and sustainable development, hydrogen has emerged as an ideal energy carrier to mitigate fossil fuel shortages and alleviate environmental concerns, due to its high energy density and zero-emission combustion products [1,2,3]. The large-scale application and industrialization of hydrogen energy rely on efficient and stable technologies for hydrogen production, purification and transportation. In this context, hydrogen separation and purification technologies directly determine the overall efficiency and economic feasibility of hydrogen energy systems and have become a core bottleneck restricting their large-scale industrial deployment. As an advanced hydrogen separation technology, PCEHPs possess unique advantages over conventional methods such as pressure swing adsorption and cryogenic separation, including low energy consumption and environmental friendliness, thus showing great application potential in high-efficiency hydrogen purification [4,5,6,7].

Perovskite-type proton conductors are promising electrolyte candidates for PCEHPs owing to their low proton migration energy and excellent proton conduction performance. As a state-of-the-art high-temperature proton ceramic electrolyte, BaZr_0.1_Ce_0.7_Y_0.1_Yb_0.1_O_3−δ_ (BZCYYb) has garnered widespread attention due to its high proton conductivity and favorable chemical stability [8,9,10]. Nevertheless, conventional BZCYYb electrolyte generally requires high operating temperatures to realize efficient proton transport. Such high-temperature operation increases energy consumption, raises requirements for thermal stability of auxiliary components, and limits its feasibility in portable and low-temperature hydrogen separation systems. Accordingly, enhancing the proton conductivity and structural stability of BZCYYb electrolytes within the intermediate–low temperature range (200–400 °C) and reducing the operating temperature are critical for the practical deployment of related materials and devices.

The transport properties of BaCeO_3_-based materials are strongly governed by A-site Ba excess. Previous studies have proven that appropriate A-site Ba excess can effectively boost proton conduction [11,12]. Jin et al. investigated the effects of Ba nonstoichiometry on the phase composition, microstructure, chemical stability and electrical conductivity of Ba_x_Ce_0.7_Zr_0.1_Y_0.1_Yb_0.1_O_3−δ_ proton conductors with 0.9 ≤ x ≤ 1.1. Grain size rose with elevated Ba excess, and the unit cell volume expanded from 333.6588 Å^3^ at x = 0.9 to 334.3041 Å^3^ at x = 1.05. In addition, higher Ba content contributed to enhanced conductivity and reduced activation energy of the samples [13]. The aforementioned studies indicate that A-site Ba non-stoichiometry can effectively enhance the proton conductivity of perovskite electrolytes, thereby holding great promise for improving the hydrogen permeation performance of PCEHPs. Nevertheless, the hydrogen-permeation behavior and practical performance of Ba-excess perovskite electrolytes under the actual operating conditions of PCEHPs remain insufficiently investigated, and relevant experimental data are still scarce. Therefore, systematic experimental studies are urgently needed to address this gap.

Apart from electrolyte materials, the structural design of hydrogen pumps also directly determines their practical performance. Conventional planar hydrogen pumps involve cumbersome fabrication procedures and repeated sintering steps, which not only incur high costs but also hinder large-scale applications [14,15]. Multilayer micro-tubular hydrogen pumps fabricated via one-step co-spinning and co-sintering can greatly simplify the manufacturing process. Meanwhile, they exhibit advantages such as high packing density and facile high-temperature sealing, serving as a crucial strategy for promoting the practical application of hydrogen pumps [16].

In this work, a series of B_x_CZYYb electrolytes with different levels of A-site Ba excess were synthesized by the solid-state reaction method. Multilayer microtubular PCEHPs were further prepared via a triple-layer one-step co-spinning and co-sintering route, with the aim of improving the intermediate–low temperature operational performance. Hydrogen separation tests were conducted within 200–400 °C to clarify the influence of Ba excess on the performance of multilayer microtubular PCEHPs. This work is expected to provide new references and an experimental basis for the design and development of high-performance proton ceramic electrochemical hydrogen pumps.

## 2. Experimental

### 2.1. Materials

The raw materials for synthesizing B_x_CZYYb powders (x = 1.05, 1.10, 1.15) included BaCO_3_, ZrO_2_, CeO_2_, Y_2_O_3_, and Yb_2_O_3_ (all 99.9% purity), purchased from Shanghai Haohong Biomedical Technology Co., Ltd. Nickel oxide (NiO, Hansi Chemical Co., Ltd., Shanghai, China) was adopted as the material for the current-collecting layer and electrodes. N-methyl-2-pyrrolidone (NMP, Yuanye Bio-Technology Co., Ltd., Shanghai, China) served as the solvent, polysulfone (PSF, Udel^®^ P-3500, Solvay, Alpharetta, GA, USA) as the polymer binder, and polyvinylpyrrolidone (PVP K90, Boai NKY Pharmaceuticals Co., Ltd., Jiaozuo, Henan, China) as the viscosity regulator. Commercial graphite powder (325 mesh, Aladdin Biochemical Technology Co., Ltd., Shanghai, China) was used as a pore-forming agent for the current-collecting layer. Terpineol (Aladdin Biochemical Technology Co., Ltd., Shanghai, China) acted as the solvent for the cathode suspension, and ethyl cellulose (Aladdin Biochemical Technology Co., Ltd., Shanghai, China) was added to tailor the slurry rheological properties.

### 2.2. Preparation of B_x_CZYYb (x = 1.05, 1.10, 1.15) Powder

B_x_CZYYb powders were synthesized via a conventional solid-state reaction method. Stoichiometric raw materials including BaCO_3_, CeO_2_, ZrO_2_, Y_2_O_3_, and Yb_2_O_3_ were accurately weighed and mixed in a planetary ball mill (XQM-4L, Changsha Tianchuang Powder Technology Co., Ltd., Hunan, China). The mixture was milled at 350 rpm for 6 h with zirconia balls as the grinding medium and ethanol as the dispersing solvent. The obtained slurry was fully dried at 90 °C and subsequently sintered in a muffle furnace at 1000 °C for 5 h under a static air atmosphere. The sintered powders were ball-milled again for 6 h and sieved through a 200-mesh screen to acquire fine B_x_CZYYb powders for the fabrication of multilayer microtubes.

### 2.3. Fabrication of Multilayer Ni/Ni-B_x_CZYYb/B_x_CZYYb/Ni-B_x_CZYYb Microtubes

Triple-layer hollow microtubes were fabricated via a co-spinning and co-sintering route. PSF and PVP were first dissolved in NMP at different ratios to prepare three independent polymer solutions for spinning. Subsequently, NiO and B_x_CZYYb ceramic powders were incorporated into each polymer solution in specific proportions, forming homogeneous suspensions for the inner current-collecting layer, anode layer, and outer electrolyte layer, respectively. Meanwhile, a certain amount of graphite powder was added to the current-collecting layer to enhance porosity. The detailed compositions of the precursor dope solutions and spinning parameters for the triple-layer precursors are summarized in Table 1.

The three spinning suspensions were continuously stirred for 72 h to achieve uniform powder dispersion, and then transferred into individual stainless steel syringes, followed by vacuum degassing for 5 h. A high-pressure syringe pump (Harvard, PHD ULTRA^TM^ 4400, Harvard Apparatus, Holliston, MA, USA) was used to precisely control the feeding rate. The three dopes were co-extruded together with deionized water as the internal coagulation medium through a custom four-orifice spinneret (φ = 1.2/2.3/3.8/4.3 mm). An air gap of 13 cm was set to guarantee adequate interlayer bonding of triple-layer precursors before entering the external coagulation bath. Finally, the freshly spun precursors were soaked in tap water to complete the phase inversion process.

After the precursor fibers were fully cured, they were cut into 36 cm segments and dried at room temperature. For cathode slurry preparation, ethyl cellulose was first stirred and fully dissolved in terpineol, and 60 wt% NiO–ceramic composite powder was then added. After homogeneous mixing, the obtained slurry was coated on the outer surface of the precursors to form the cathode layer. The dried four-layer microtube precursors were sintered in a box-type furnace at 1425 °C for 300 min in an air atmosphere to realize full densification of the electrolyte layer.

### 2.4. Hydrogen Separation Performance Test

**Figure 1:**
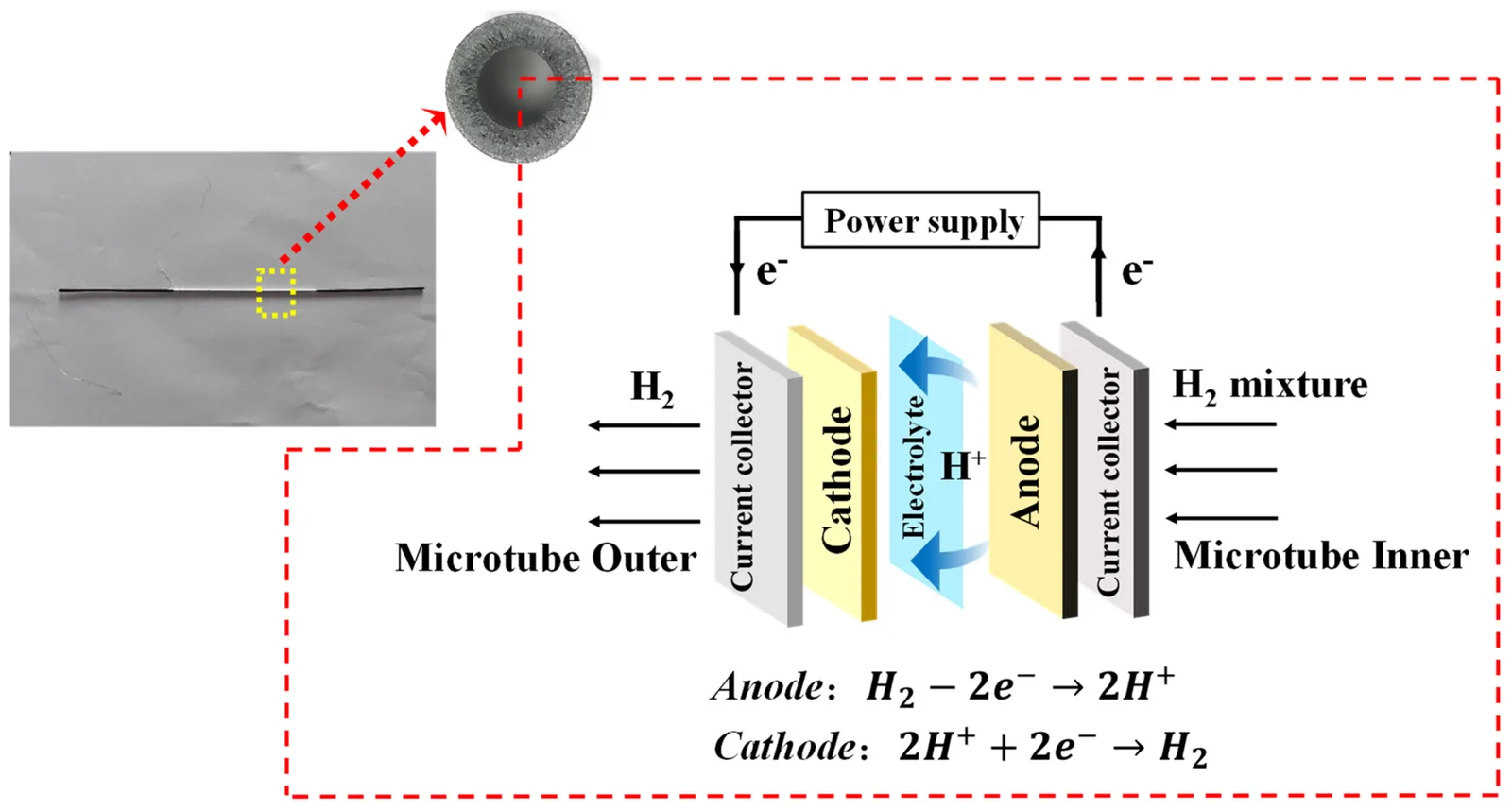
Schematic of the multilayer proton ceramic microtube.

For electrochemical performance measurements, silver paste was coated on the cathode surface, and silver wires were attached to both inner and outer surfaces as current leads. The multilayer microtubes were then sintered at 800 °C for 20 min to eliminate organic residues and secure the silver paste. The test system was sealed with high-temperature ceramic adhesive to achieve gas-tight isolation between the anode and cathode chambers and prevent gas cross-leakage during electrochemical measurements. A schematic diagram of the multilayer microtube structure is presented in Figure 1. After reduction, the air tightness of the multilayer microtubes was tested by a self-designed nitrogen permeation device, whose structure is shown in Figure S1. The measured nitrogen permeation flux was 1.17 × 10^−8^ mol m^−2^ s^−1^ Pa^−1^ under a pressure difference of 0.35 MPa. All reduction and subsequent tests were carried out under low oxygen partial pressure. Gas chromatography was used to continuously monitor the oxygen concentration in the gas phase to maintain a stable reducing atmosphere and prevent the re-oxidation of metallic nickel. The assembled samples were reduced at 700 °C in a pure H_2_ atmosphere for 5 h to fully reduce NiO to metallic Ni. The electrochemical hydrogen pump test rig was referenced from our group’s previous work [17], and hydrogen pumping performance was evaluated within 200–400 °C. During the test, the anode was fed with a dry H_2_/He mixed gas containing 30 vol% H_2_ at a flow rate of 50 mL min^−1^, while pure dry H_2_ was supplied to the cathode at 50 mL min^−1^. The flow rates of feed and purge gases were regulated by mass flow controllers (Beijing Sevenstar Flow Co., Ltd., Beijing, China) and calibrated with a bubble flow meter. A direct voltage was applied to the multilayer microtubes via a source meter (Keithley 2440, Keithley Instruments, Solon, OH, USA). A constant DC voltage ranging from 0.5 to 2 V was applied for electrochemical measurements. Data collection was initiated only after the current remained stable for more than 5 min. The outlet gas composition was analyzed by a gas chromatograph (Agilent 7890B, Agilent Technologies, Santa Clara, CA, USA). The hydrogen permeation flux was calculated by the following equation:

(1) JH2=FoutCH2−CHefH2fHe−FinS

where

(2) S=πDo−DiLlnDoDi

In this formula, S is defined as the effective permeation area of the membrane (cm^2^). D_o_ and D_i_ represent the outer and inner membrane diameters (cm), while L means the valid membrane length fixed at 5 cm, consistent with the constant heating zone of the testing furnace. F_in_ and F_out_ are the inlet and outlet gas flow rates on the permeate side, with units of mL min^−1^. The parameters C_H2_ and C_He_ describe the respective gas concentrations in the outlet stream, and f_H2_ and f_He_ stand for the volume fractions of these two components in the raw feed gas.

The theoretical hydrogen pumping flux can be calculated using Equation (3):

(3) $$J=\frac{1344i}{\mathrm{nF}}$$

where J denotes the hydrogen flux in units of mL min^−1^ cm^−2^; i represents the current density with the unit mA cm^−2^; *n* refers to the number of electrons transferred in the electrochemical reaction, and *n* = 2 for hydrogen evolution; F stands for the Faraday constant, which takes a value of 96,485.33 C/mol.

### 2.5. Characterizations

The microstructure of reduced Ni-B_x_CZYYb/B_x_CZYYb/Ni-B_x_CZYYb/Ni multilayer microtubes was characterized by scanning electron microscopy (SEM, FEI NOVA Nano 450, Hillsboro, OR, USA). The phase composition of the as-prepared materials was analyzed via X-ray diffraction (XRD, BRUKER D8 ADVANCE, Karlsruhe, Germany) with Cu Kα radiation (λ = 1.5418 Å) at 40 kV and 40 mA. For electrical conductivity tests, B_x_CZYYb powders with different levels of Ba excess were compacted into rectangular pellets in a stainless steel mold (2.5 cm × 1 cm × 0.3 cm) and sintered at 1430 °C. Silver paste was coated on both sides of the sintered pellets and then heat-treated at 800 °C for 20 min under static air conditions. Electrochemical impedance spectroscopy (EIS) was performed on a Gamry electrochemical workstation.

## 3. Results and Discussion

### 3.1. Microstructure and Phase Structure Characterization

**Figure 2:**
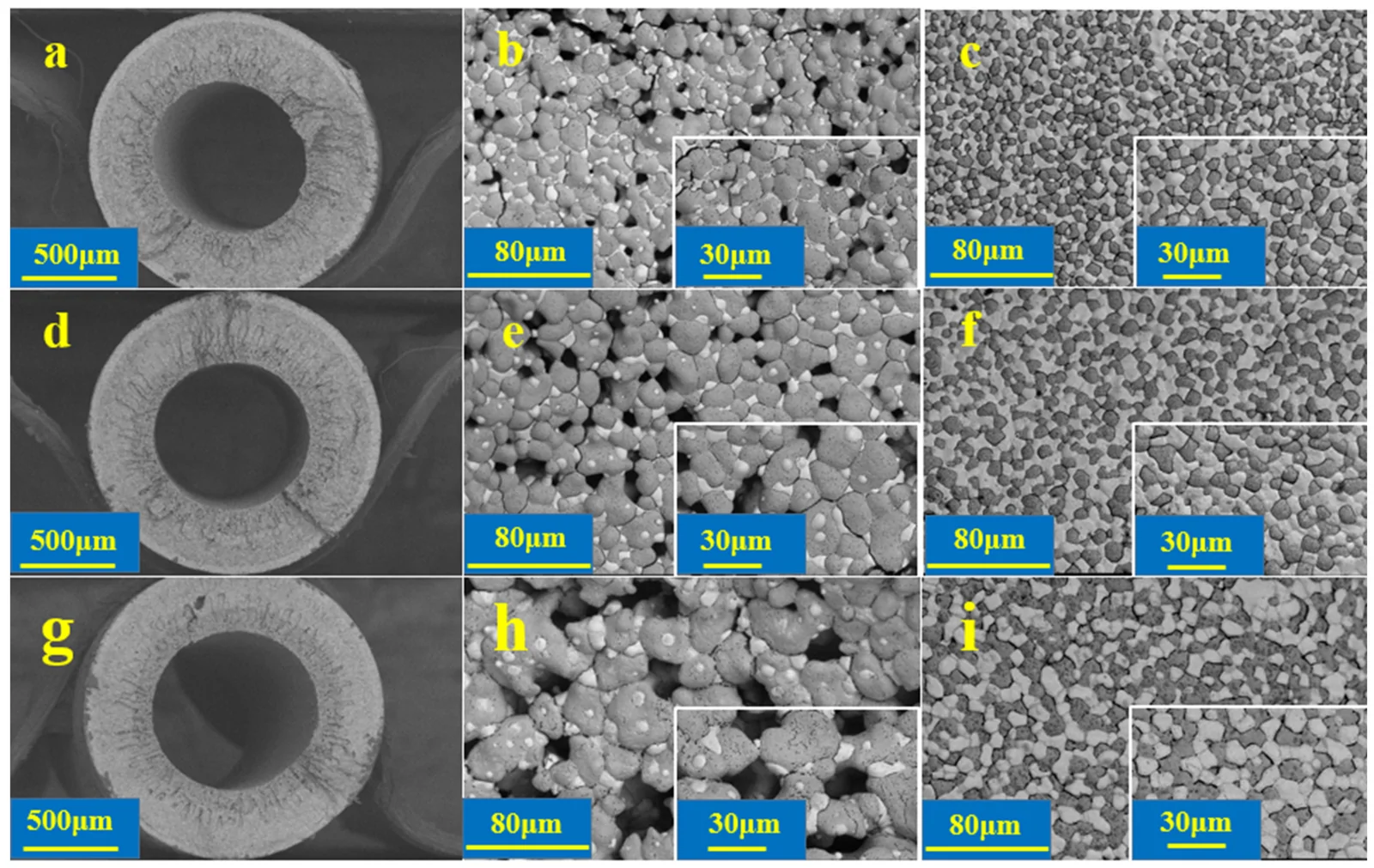
Microscopic morphology of reduced multilayer microtubes with different Ba excess levels (x = 1.05, 1.10, 1.15). (**a**,**d**,**g**) Cross-sectional morphology; (**b**,**e**,**h**) Inner surface morphology; (**c**,**f**,**i**) Outer surface morphology.

The microstructure of reduced multilayer Ni/Ni-B_x_CZYYb/B_x_CZYYb/Ni-B_x_CZYYb microtubes is presented in Figure 2. The interfacial bonding and surface morphology of each functional layer directly govern the hydrogen separation capacity of the electrochemical hydrogen pump. Figure 2a,d,g show the cross-sectional SEM views of samples with a Ba excess of 1.05, 1.10 and 1.15, respectively. The overall thicknesses of these multilayer microtubes are 360 μm, 370 μm and 365 μm in order. The overall cross-section shows no obvious cracks at the interfaces among the cathode, electrolyte, anode, and inner current-collecting layer, which demonstrates good interfacial compatibility of each layer after reduction treatment. This favorable structural feature stems from the optimized slurry composition and spinning conditions, which effectively alleviate structural defects caused by interlayer thermal stress. In addition, finger-like pores generated during phase inversion can lower the resistance of gas diffusion.

Figure 2b,e,h shows the inner surface morphologies of microtubes with a Ba excess of 1.05, 1.10 and 1.15, respectively. After high-temperature sintering, graphite in the inner current-collecting layer forms interconnected porous channels. This structure greatly reduces hydrogen diffusion resistance from the inner wall to functional layers and enhances overall gas mass transfer efficiency. Figure 2c,f,i presents the outer surface microstructures of samples with different Ba excess levels. The porous architecture derived from NiO high-temperature reduction favors high catalytic activity. Meanwhile, the degree of Ba excess exerts a prominent influence on the microscopic morphology. As shown in Figure S2, a large number of white impurity precipitates are observed on the grain surface of the electrolyte layer in multilayer microtubes when B_1.15_CZYYb is adopted as the electrolyte. Combined with the EDS line-scan (Figure S3), spot-scan results (Figure S4) and elemental analysis in Table S1, these white particles are Ni-rich. Given that Figure S2 was obtained after reduction treatment, they are tentatively assigned to metallic Ni. According to the crystal-structure characteristics of perovskite, excessive A-site Ba may induce severe lattice distortion [18], which could reduce the solid-solubility limit of Ni ions in the perovskite lattice. Consequently, Ni might segregate and accumulate on grain surfaces. Since Ni cannot provide pathways for proton conduction, the proton-conduction efficiency of the material may be degraded.

**Figure 3:**
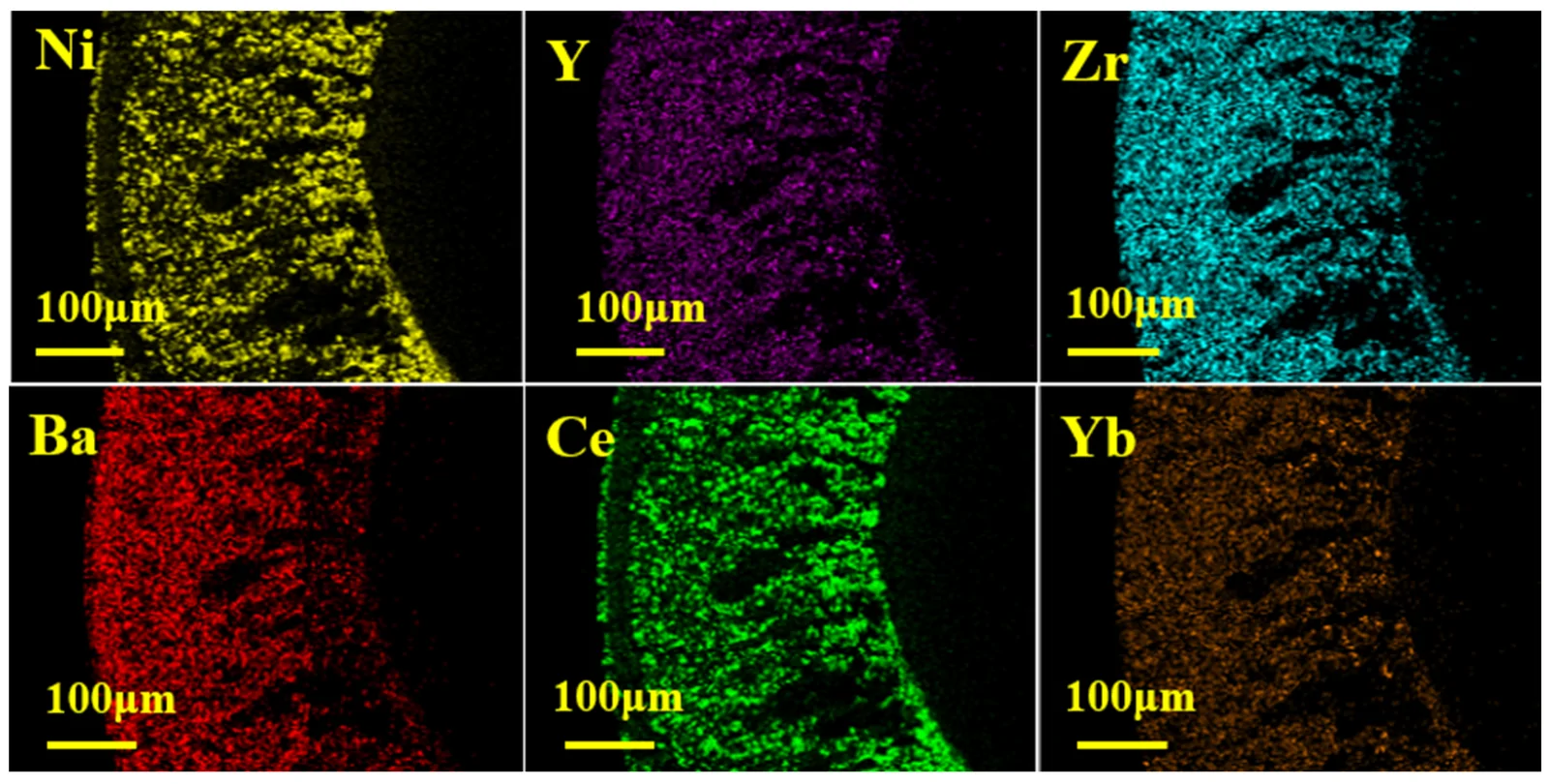
EDS Elemental Mapping Analysis of Cross-Sectional Multilayer Microtubes with a Ba excess ratio of 1.05.

Multilayer proton ceramic microtubes are fabricated via a high-temperature co-sintering route. During sintering, metallic Ni contained in the anode and cathode readily undergoes interlayer diffusion [19]. Although slight Ni diffusion toward the electrode-electrolyte interface can improve electrolyte sinterability and facilitate the formation of dense electrolyte substrates, extensive Ni penetration into the electrolyte drastically increases the overall electronic conductivity and triggers electronic leakage, which severely impairs the separation efficiency of hydrogen pumps. Figure 3 presents the EDS elemental mapping of multilayer microtubes with a Ba excess of 1.05 after high-temperature sintering. No distinct Ni signals are detected within the electrolyte region; Ni is only distributed in the anode and cathode functional layers. This confirms that the preparation procedure adopted in this work can effectively suppress Ni interlayer diffusion, guaranteeing exclusive proton conduction in the electrolyte layer and eliminating parasitic electronic conduction losses.

**Figure 4:**
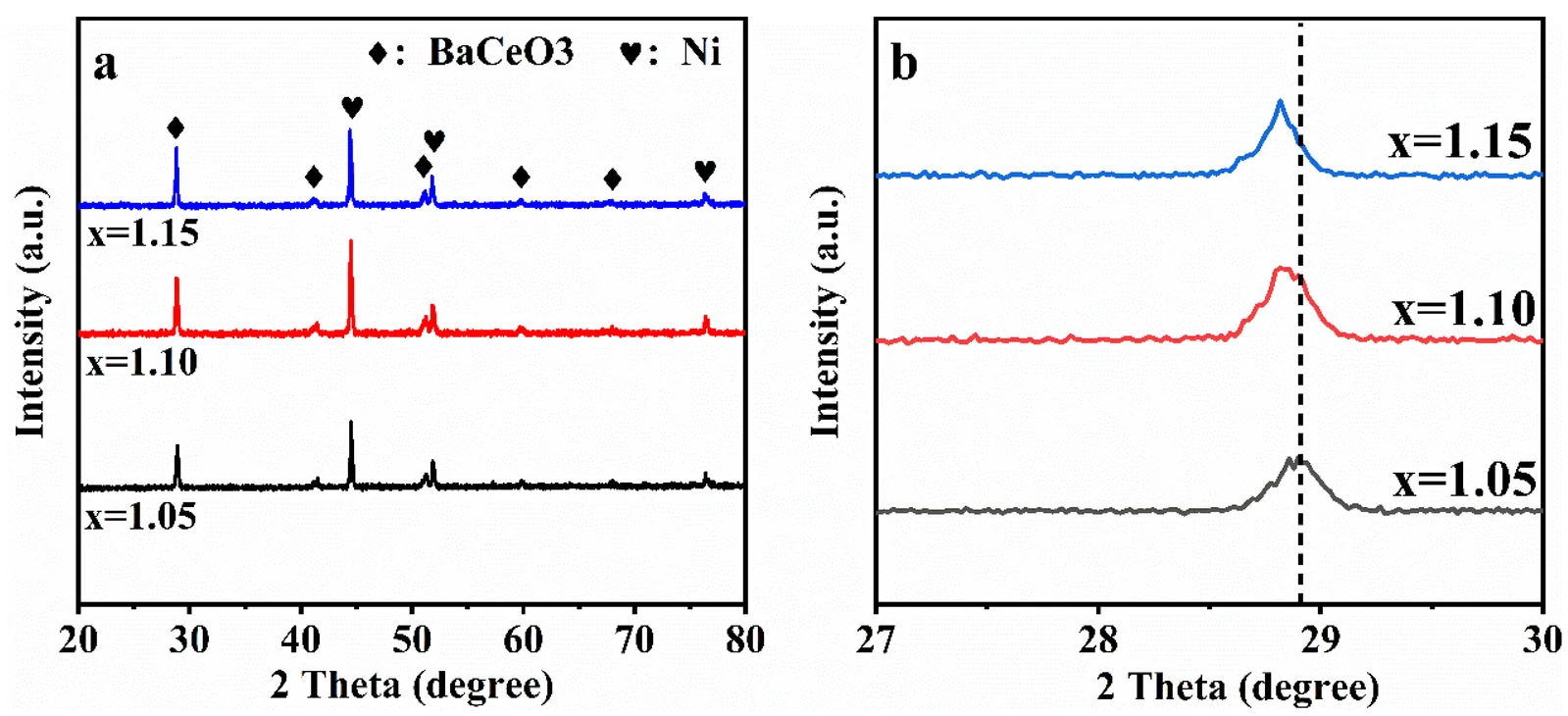
(**a**) XRD profiles of B_x_CZYYb multilayer microtubes with varying Ba excess levels after post-sintering reduction; (**b**) Magnified diffraction patterns in the range of 27° to 30°.

XRD tests were performed on microtube samples after high-temperature sintering and reduction at 700 °C. As shown in Figure 4a, only two crystalline phases were detected in the multilayer hydrogen pumps with three different Ba excess. One is a typical cubic perovskite structure with the space group Pm3m, which is well matched with the standard BaCeO_3_ PDF card (PDF#75-0431). The other is assigned to metallic Ni (PDF#87-0712). The existence of pure metallic Ni endows the inner current-collecting layer, anode and cathode layers with excellent electronic conductivity. Figure 4b shows the magnified XRD patterns in the diffraction range of 27–30°. As shown by the results, the unit-cell volume of the BaCeO_3_ phase exhibits a significant increasing trend as the Ba-excess level rises in the multilayer co-sintered system. This variation trend is consistent with previous studies [20,21,22]. The calculated unit cell volumes for x = 1.05, 1.10 and 1.15 are 83.22, 83.45 and 83.76 Å^3^, respectively. Furthermore, to explore the feasibility of studying the effect of Ba excess independently without NiO interference, single-layer B_1.05_CZYYb microtubes with and without NiO addition were prepared and sintered at 1500 °C. Although both samples form a crystalline perovskite structure (Figure S5), they exhibit obvious differences in macroscopic appearance (Figure S6). Gas-tightness was examined using a home-built testing setup. High-pressure gas was introduced into the interior of microtubes, which were then submerged in anhydrous ethanol for observation. As shown in Figure S7, abundant interconnected open pores were observed in NiO-free microtubes sintered at 1500 °C. Accordingly, the NiO-free samples exhibit extremely poor gas-tightness and cannot be used for subsequent performance measurements. This is also reflected in their linear shrinkage: after sintering at 1500 °C, the NiO-free sample exhibits a linear shrinkage of approximately 23.8%, while the NiO-containing sample shows a linear shrinkage of around 32.5%.

### 3.2. Hydrogen Pump Performance Evaluation

To further explore the influence of Ba excess on hydrogen pump performance, a 30 vol% H_2_–He mixture was introduced into the anode at a flow rate of 50 mL min^−1^, while pure hydrogen was delivered to the cathode as purge gas at 50 mL min^−1^. Electrochemical behaviors of hydrogen pumps with B_x_CZYYb electrolytes (x = 1.05, 1.10, 1.15) were systematically tested at 200–400 °C under an applied voltage of 2 V. The outlet flow rate on the cathode side was recorded with a bubble flow meter, and the hydrogen permeation flux was calculated, based on outlet gas composition analyzed via gas chromatography.

**Figure 5:**
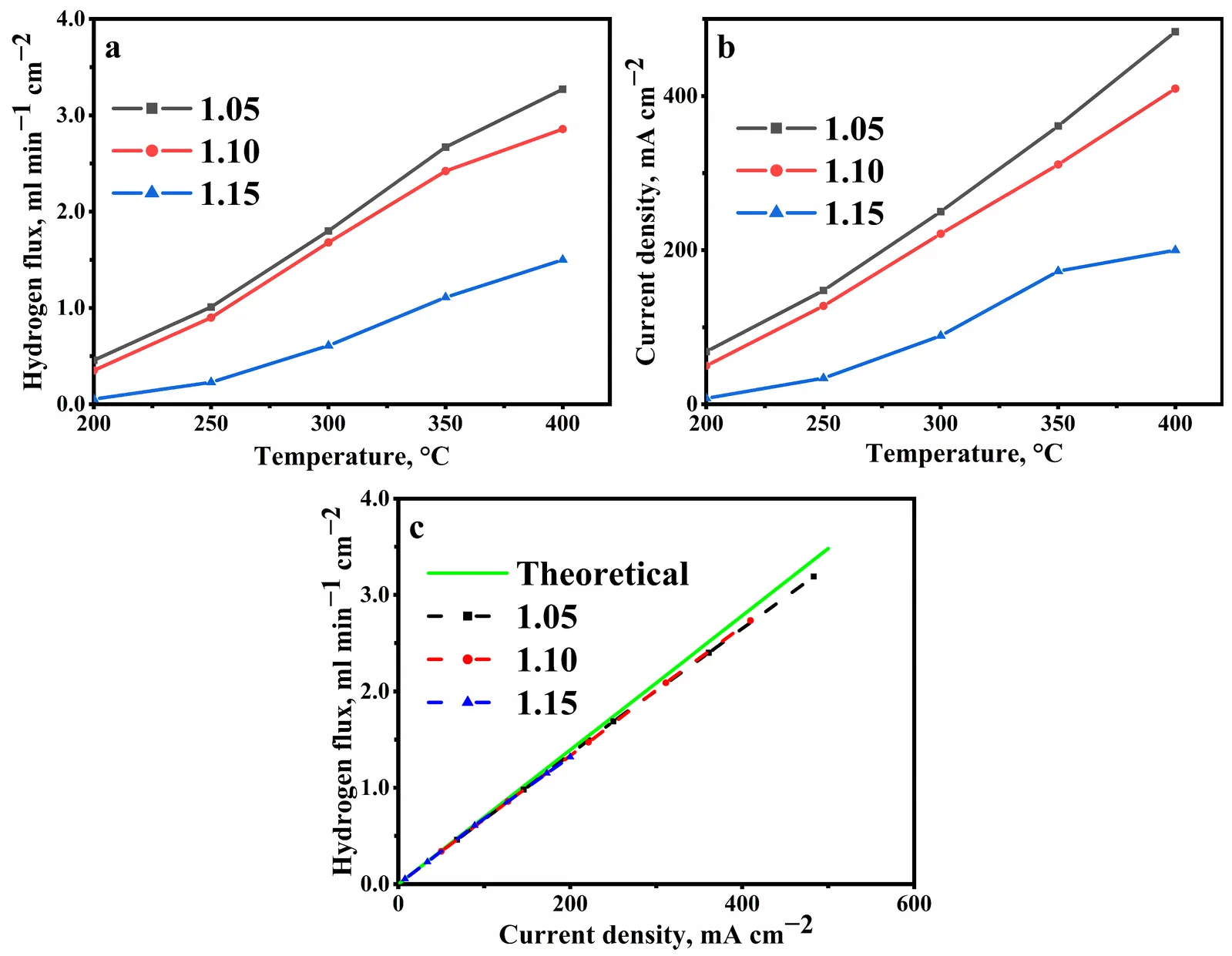
Electrochemical performance of B_x_CZYYb microtubular hydrogen pumps with varying Ba excess levels (x = 1.05, 1.10, and 1.15). (**a**) Experimental hydrogen permeation flux as a function of temperature (200–400 °C) under an applied voltage of 2 V; (**b**) Corresponding current density as a function of temperature at 2 V; (**c**) Measured hydrogen flux versus measured current density under 2 V, where the green line denotes the theoretical hydrogen flux calculated by Faraday’s law (assuming 100% Faradaic efficiency).

Figure 5a shows the temperature dependence of hydrogen permeation flux for electrochemical hydrogen pumps with different Ba excess levels at a constant applied voltage of 2 V. The sample with the B_1.05_CZYYb electrolyte delivers the best hydrogen permeation performance across the entire test temperature range. At 400 °C, the hydrogen permeation flux of the x = 1.05 sample reaches 3.19 mL min^−1^ cm^−2^. As the Ba excess increases to x = 1.10, the flux decreases slightly by 14% compared with the x = 1.05 group. A further increase in Ba excess to x = 1.15 leads to a distinct deterioration in hydrogen permeation flux, with a prominent reduction of 53%. A comprehensive performance comparison between the hydrogen pump fabricated in this work and state-of-the-art devices reported in the previous literature is summarized in Table S2, which verifies the outstanding competitiveness of our as-prepared microtubular hydrogen pump. In general, NiO as a sintering aid can improve the sinterability by forming transient BaNiO_3_ or BaY_2_NiO_5_ solid solutions, which increases the unit-cell volume and reduces the number of grain boundaries. Meanwhile, NiO may substitute for B-site Ce ions in the perovskite lattice to induce the formation of oxygen vacancies and thus enhance proton conduction [23,24]. However, excessive A-site Ba can further expand the unit-cell volume and aggravate lattice distortion, weakening the solid-solution capacity of the perovskite lattice for Ni. Once Ni exceeds the lattice solid-solubility limit, Ni may migrate to grain surfaces and accumulate to form impurity precipitates.

For BaCeO_3_-based perovskite electrolytes, the Ce^4+^/Ce^3+^ redox reaction under high temperature and applied electric field induces obvious electronic conduction [25,26]. Such electronic conduction competes with proton transport, reduces the Faradaic efficiency of hydrogen pumps, and raises energy consumption. In contrast, operation within the medium–low temperature range can effectively suppress electronic leakage and maintain high Faradaic efficiency. As shown in Figure 5b, the current density of all microtubular hydrogen pumps increases significantly with temperature in the range of 200–400 °C under a fixed applied voltage, while the samples with moderate Ba excess (x = 1.05) consistently exhibit the highest values, indicating optimal electrochemical performance. As presented in Figure 5c, all microtubular hydrogen pumps with different Ba non-stoichiometric excess exhibit outstanding Faradaic efficiencies, which remain steadily above 95% even at relatively high current densities. This result confirms that the designed electrolyte composition and suitable operating temperature effectively restrain electronic conduction, enabling proton conduction to become the dominant transport process. Consequently, high-efficiency and low-energy-consumption hydrogen separation can be realized.

### 3.3. Electrical Conductivity Characterization

**Figure 6:**
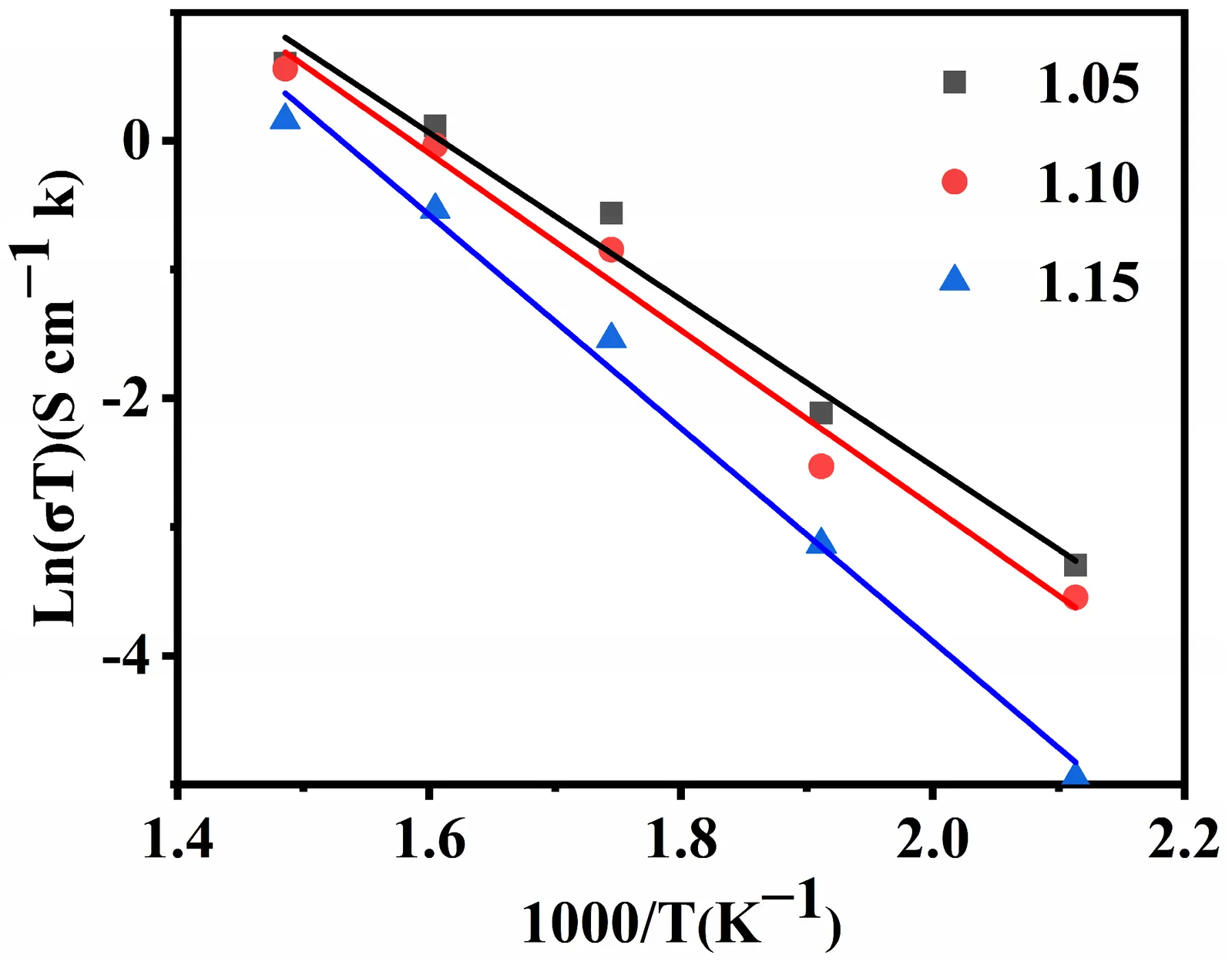
Temperature-dependent electrical conductivity of B_x_CZYYb with x = 1.05, 1.10 and 1.15 in 10 vol% H_2_/N_2_ atmosphere.

To replicate the actual co-sintered environment of the electrolyte layer in multilayer microtubes, a certain amount of NiO was introduced during the preparation of bulk electrolyte materials. The influence of Ba excess on the electrical conductivity of B_x_CZYYb (x = 1.05, 1.10, 1.15) was evaluated under a dry atmosphere consisting of 10 vol% H_2_ balanced with N_2_. As shown in Figure 6, the conductivity–temperature curves of all samples rise steadily, displaying typical thermally activated conduction characteristics. At identical temperatures, the B_1.05_CZYYb sample maintains the highest total conductivity, which is in good agreement with previous reports [27]. For the optimal B_1.05_CZYYb sample, conductivity measurements were performed at 200–400 °C under atmospheres with multiple gradient hydrogen partial pressures, and the test results are presented in Figure S8. The plotted curves demonstrate that hydrogen partial pressure strongly modulates the proton conductivity of B_1.05_CZYYb, which rises synchronously with increasing hydrogen partial pressure. At the identical temperature, the conductivity follows the order: 60 vol% H_2_ > 30 vol% H_2_ > 10 vol% H_2_. A high hydrogen partial pressure raises the concentration of proton defects in the perovskite lattice, increases the quantity of mobile proton carriers in the system, and ultimately improves the proton conduction performance. According to the Arrhenius equation σT = Aexp(−E_a_/RT) (where σ is the conductivity, A is the pre-exponential factor, T is the absolute temperature, R is the universal gas constant, and E_a_ is the activation energy), the conduction activation energies of the B_1.05_CZYYb, B_1.10_CZYYb and B_1.15_CZYYb samples in the medium–low temperature range were calculated to be 54, 57, and 69 kJ mol^−1^, respectively.

### 3.4. Electrochemical Impedance Analysis

**Figure 7:**
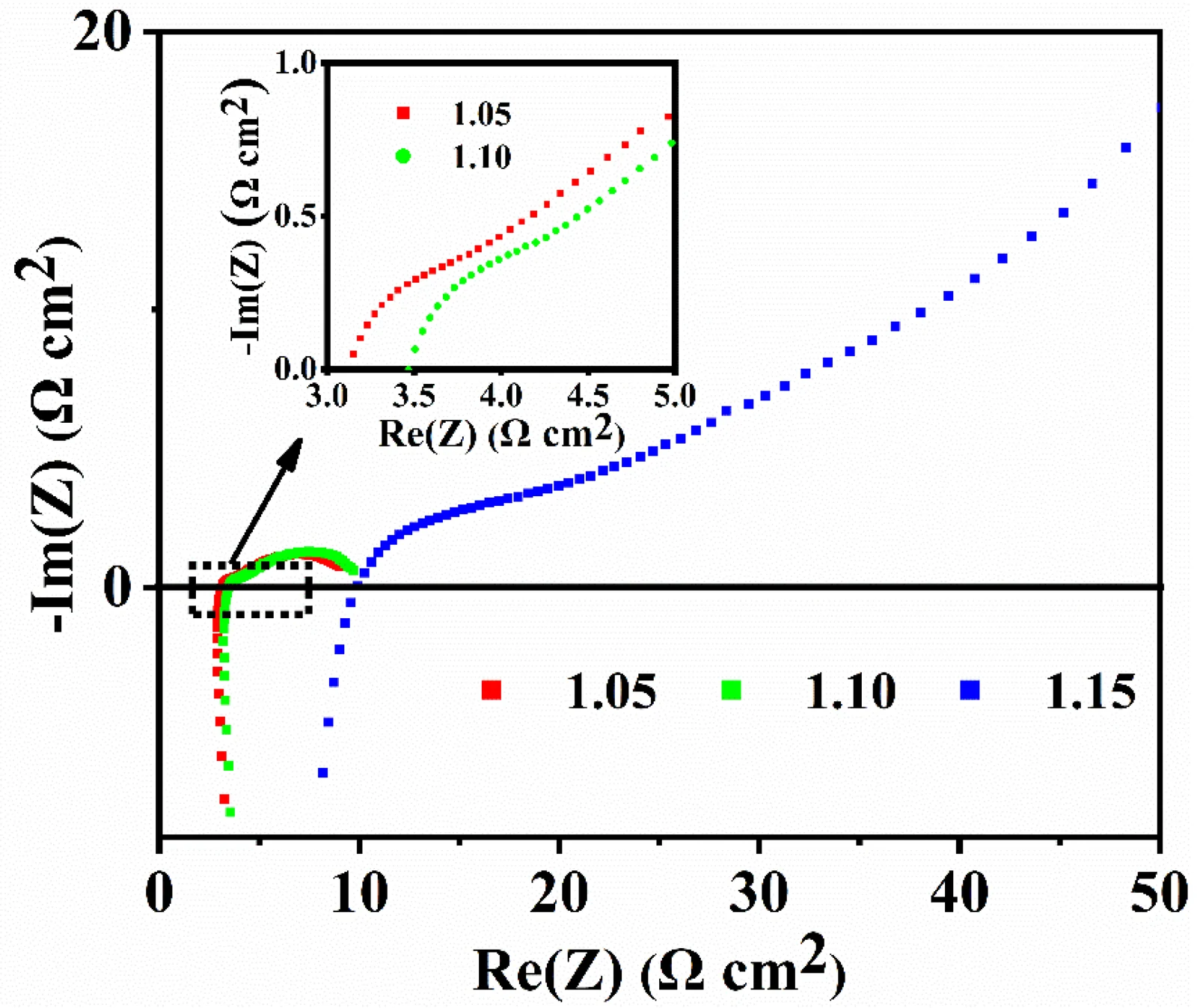
Nyquist EIS spectra of microtubular hydrogen pumps with different Ba excess levels measured at 300 °C under OCV.

The hydrogen pump was tested under the same atmosphere as the hydrogen separation experiments, and electrochemical impedance spectra were collected at open-circuit voltage. As shown in Figure 7, the ohmic resistance of multilayer proton ceramic electrochemical hydrogen pumps continuously increases with rising Ba excess from x = 1.05 to 1.15. The fitted ohmic resistance values are 3.0, 3.3 and 10.8 Ω, respectively, as summarized in Table S3 and Figure S9. Under identical test conditions, the ohmic resistance (Rs) of the device increases markedly with growing Ba excess. This may be attributed to Ni segregation induced by excessive Ba doping. Although the B_1.05_CZYYb sample exhibits the minimum ohmic resistance, its corresponding assembled hydrogen pump still presents prominent polarization resistance. Rational optimization of the electrode pore structure and interfacial catalytic activity can effectively reduce polarization loss, which further improves the hydrogen permeation capacity of the device.

## 4. Conclusions

In this work, multilayer microtubular protonic ceramic electrochemical hydrogen pumps (PCEHPs) with B_x_CZYYb electrolytes of varying Ba-excess levels (x = 1.05, 1.10, 1.15) were fabricated by a one-step co-spinning and co-sintering method, and the B_1.05_CZYYb device delivers the optimal hydrogen-separation performance. EDS results reveal Ni-rich precipitates on grain surfaces in the co-sintered electrolyte. It is speculated that excessive Ba may induce lattice distortion and reduce Ni solubility in the perovskite lattice, promoting Ni exsolution, which could in turn hinder proton conduction and increase ohmic resistance. Notably, this mechanistic interpretation is applicable only to the present Ni-containing co-sintered microtubular system; the observed unit-cell expansion, resistance variation, and Ni segregation are overall apparent behaviors of this co-sintered system and should not be extrapolated as intrinsic properties of Ni-free electrolytes. This study provides experimental and theoretical references for the composition optimization and structural design of Ni-containing co-sintered microtubular PCEHPs. Future work on targeted pore-structure regulation may further reduce mass-transfer resistance and enhance hydrogen-separation efficiency, and the device may be coupled with electrochemical CO_2_ reduction systems for high-value-added product synthesis.

## Figures and Tables

**Table 1:** Composition and Preparation Parameters of Microtube Precursors.

Suspension	Composition, wt%						Spinning Rate, mL min^−1^
	NMP	PSF	B_x_CZYYb	NiO	PVP	Graphite	
Inner layer	37.8	9.4	4.7	42.5	1.4	4.1	1.4
Middle layer	30.6	7.7	24.5	36.7	0.5	0	3.9
Outer layer	30.7	7.7	60.7	0.6	0.3	0	1.2

## Data Availability

The data that supports the findings of this study are available in the Supporting Information of this article.

## Supplementary Materials

The following supporting information can be downloaded at: https://www.mdpi.com/article/10.3390/membranes16090307/s1, Figure S1: Configuration of the self-made nitrogen permeation device used for airtightness testing. Figure S2: Cross-sectional magnified micrograph of the multilayer microtube with B_1.15_CZYYb as electrolyte after high-temperature sintering and subsequent reduction treatment. Figure S3: EDS line-scan spectra acquired from the magnified region of the electrolyte layer in the cross-section of the multilayer microtube shown in Figure S2. Figure S4: EDS spot-scan spectra obtained from the magnified cross-sectional region of the multilayer microtube in Figure S2. (1) White granular precipitate region; (2) Perovskite matrix region. Figure S5: XRD patterns of NiO-free and NiO-containing B_1.05_CZYYb electrolyte materials sintered at 1500 °C. Both patterns were normalized by setting the intensity of the main perovskite diffraction peak to unity. The curve of the NiO-containing sample is vertically offset for clarity of comparison. Figure S6: Photographs of NiO-free and NiO-containing B_1.05_CZYYb microtubes sintered at 1500 °C. Left: NiO-free samples; Right: NiO-containing samples. (After sintering at 1500 °C, the NiO-free sample exhibits a linear shrinkage of approximately 23.8%, while the NiO-containing sample shows a linear shrinkage of around 32.5%. Figure S7: Gas-tightness test for the NiO-free microtube sintered at 1500 °C, where high-pressure gas was fed into the inner cavity of the microtube. Figure S8: Temperature dependence of proton conductivity of the B_1.05_CZYYb microtubular hydrogen pump under various hydrogen concentrations (10 vol.%, 30 vol.%, 60 vol.% H_2_ balanced by N_2_). Figure S9: EIS Nyquist spectra of microtubular proton ceramic hydrogen pumps with different Ba excess levels. Subplots (a), (b), and (c) correspond to samples with Ba excess levels of x = 1.05, 1.10 and 1.15, respectively. Table S1: Mass percentages of typical elements measured by EDS spot scanning for multilayer microtube with B_1.15_CZYYb electrolyte. Table S2: Comparison of Hydrogen Pumping Performance among Recently Reported PCEHPs. Table S3: Ohmic Resistance and Polarization Resistance of Multilayer Hydrogen Pump Cells with Different Ba Excess Levels.
