# Supplementary material for: Effects of Barium Excess on the Performance of Multilayer Microtubular Proton Ceramic Electrochemical Hydrogen Pumps with BaxCe0.7Zr0.1Y0.1Yb0.1O3−δ (x = 1.05, 1.10, 1.15)
Source: Membranes (Basel). 2026 Sep 20;16(9):307. doi: 10.3390/membranes16090307 (PMC13609753; doi:10.3390/membranes16090307)
Supplement: Supplementary file 1 [file membranes-16-00307-s001.zip › membranes-4321374-supplementary.pdf]

Supplementary Material

Effects of Barium Excess on the Performance of Multilayer Microtubular Proton Ceramic Electrochemical Hydrogen Pumps with $Ba_xCe_{0.7}Zr_{0.1}Y_{0.1}Yb_{0.1}O_{3-\delta}$ (x=1.05, 1.10, 1.15)

Shao Zhang [1], Lihui Wang [1], Congcong Li [1], Mingming Wang [1,2,*], Zhigang Wang [1,2,*] and Xiaoyao Tan [1,*]

[1]State Key Laboratory of Advanced Separation Membrane Materials, College of Chemical Engineering and Technology, Tiangong University, Tianjin 300387, China

[2]Hebei Industrial Technology Research Institute of Membranes, Cangzhou Institute of Tiangong University, Cangzhou 061000, China

[*]Correspondence: wangmingming@tiangong.edu.cn (M.W.); wangzhigang@tiangong.edu.cn (Z.W.); tanxiaoyao@tiangong.edu.cn (X.T.)

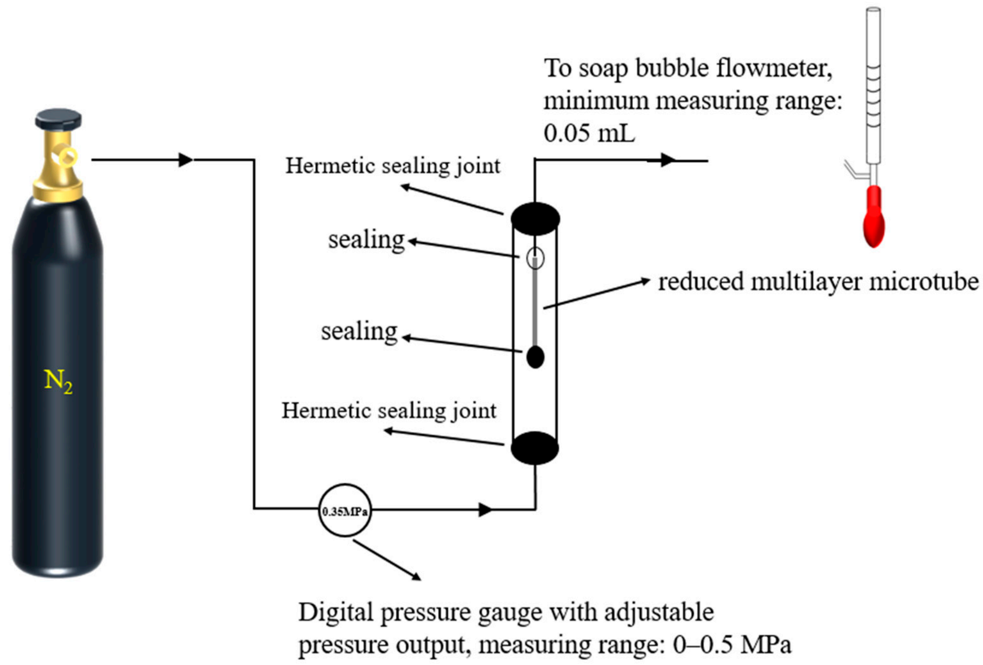

Figure S1. Configuration of the self-made nitrogen permeation device used for airtightness testing.

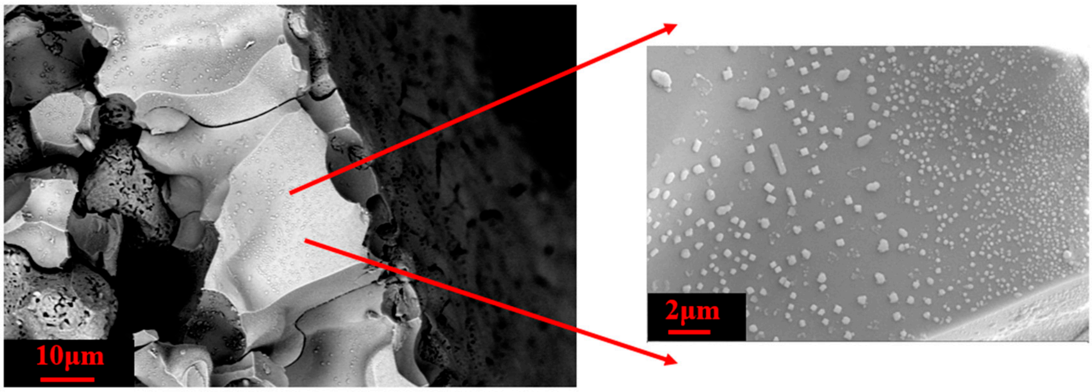

Figure S2. Cross-sectional magnified micrograph of the multilayer microtube with $B_{1.15}$CZYYb as electrolyte after high-temperature sintering and subsequent reduction treatment.

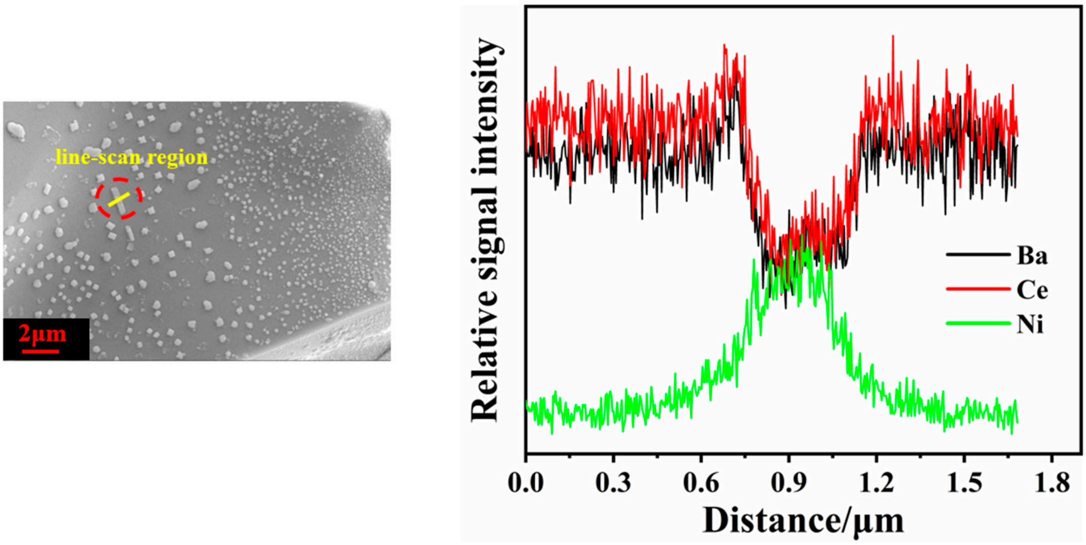

Figure S3. EDS line-scan spectra acquired from the magnified region of the electrolyte layer in the cross-section of the multilayer microtube shown in Fig. S2.

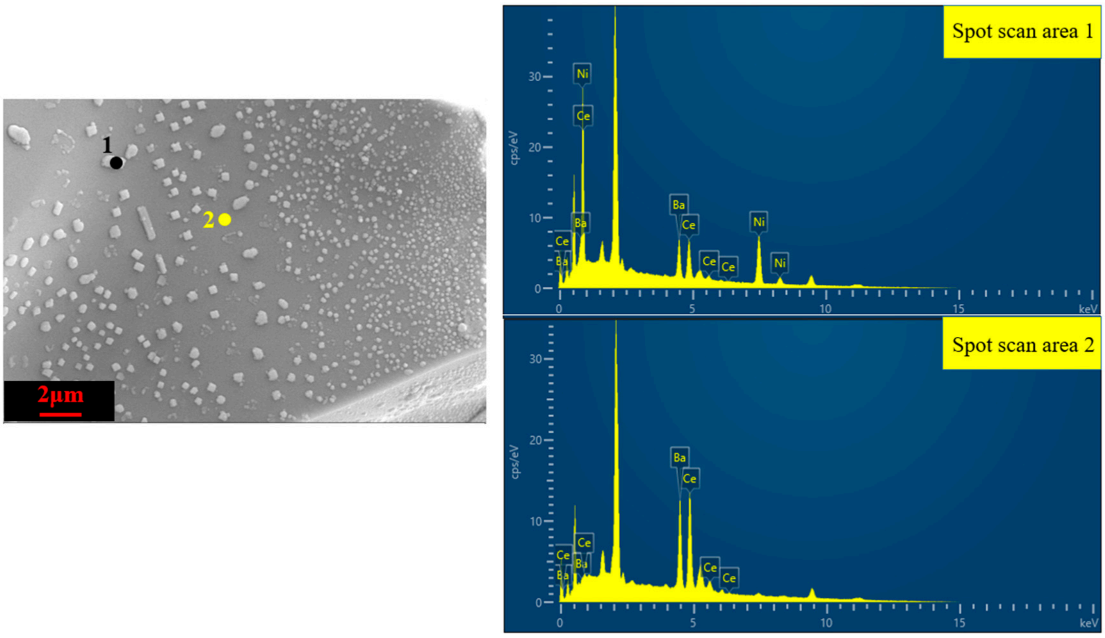

Figure S4. EDS spot-scan spectra obtained from the magnified cross-sectional region of the multilayer microtube in Fig. S2. (1) White granular precipitate region; (2) Perovskite matrix region.

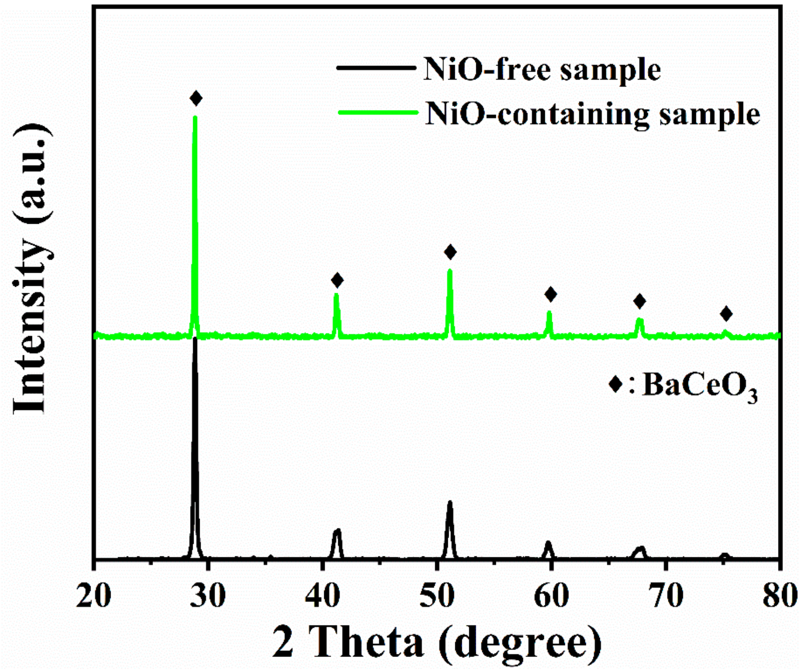

Figure S5. XRD patterns of NiO-free and NiO-containing $B_{1.05}CZYYb$ electrolyte materials sintered at 1500 °C. Both patterns were normalized by setting the intensity of the main perovskite diffraction peak to unity. The curve of the NiO-containing sample is vertically offset for clarity of comparison.

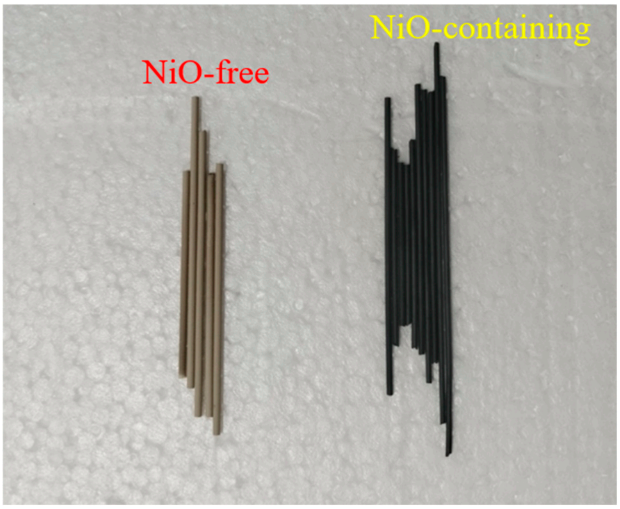

Figure S6. Photographs of NiO-free and NiO-containing $B_{1.05}CZYYb$ microtubes sintered at 1500 °C. Left: NiO-free samples; Right: NiO-containing samples. (After sintering at 1500 °C, the NiO-free sample exhibits a linear shrinkage of approximately 23.8%, while the NiO-containing sample shows a linear shrinkage of around 32.5%.

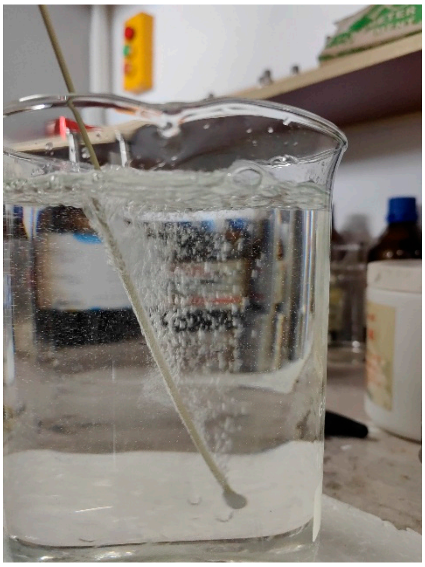

Figure S7. Gas-tightness test for the NiO-free microtube sintered at 1500 °C, where high-pressure gas was fed into the inner cavity of the microtube.

Table S1. Mass percentages of typical elements measured by EDS spot scanning for multilayer microtube with $B_{1.15}$CZYYb electrolyte

Element	Spot data 1	Spot data 2
Ba	28.94	58.55
Ni	52.58	-
Ce	18.48	41.45

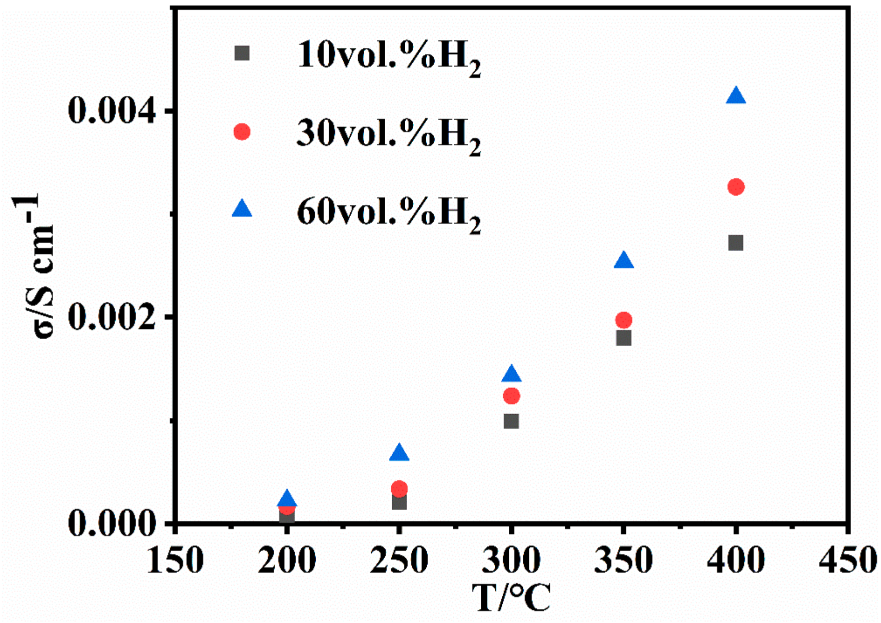

Figure S8. Temperature dependence of proton conductivity of the $B_{1.05}$CZYYb microtubular hydrogen pump under various hydrogen concentrations (10 vol.%, 30 vol.%, 60 vol.% H_2 balanced by N_2).

Fig. S9 displays the EIS Nyquist spectra of microtubular proton ceramic hydrogen pumps with different levels of Ba excess. All spectra were recorded at 300 °C under open-circuit voltage (OCV). Black diamond symbols denote experimental raw data, and solid black lines are fitting curves derived from the equivalent circuit illustrated in the inset of Fig. S9(c). Vertical red lines mark characteristic frequencies to distinguish the ohmic resistance region, interfacial polarization region and low-frequency diffusion region.

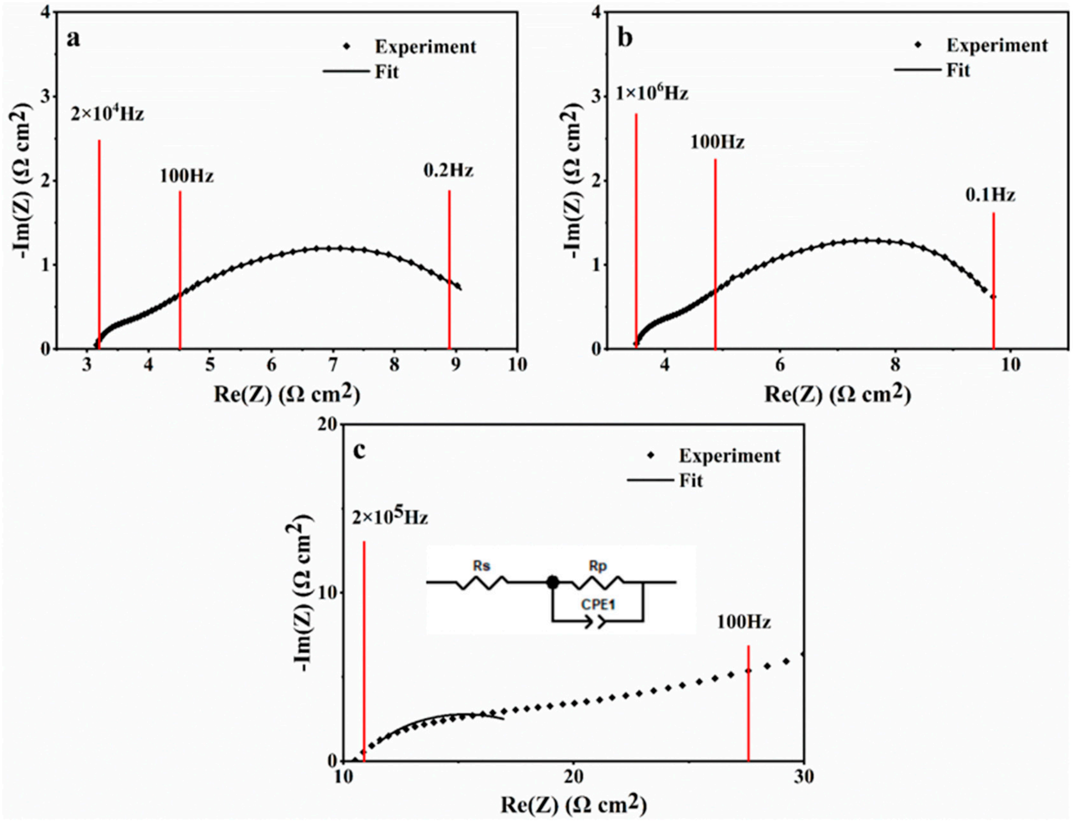

Figure S9. EIS Nyquist spectra of microtubular proton ceramic hydrogen pumps with different Ba excess levels. Subplots (a), (b), and (c) correspond to samples with Ba excess levels of x = 1.05, 1.10 and 1.15, respectively.

Table S2

Comparison of Hydrogen Pumping Performance among Recently Reported PCEHPs.

configuration	Temperature/°C	applied voltage/v	geometry	Faradaic efficiency/%	hydrogen flux/ ml min-1 cm-2	Ref.
Ni/Ni-$B_{1.05}$CZYYb/ $B_{1.05}$CZYYb/Ni- $B_{1.05}$CZYYb	200	2	microtubular	96	0.46	This work
	250			98	1.01	
	300			97	1.69	
	350			95	2.40	
	400			95	3.19	
Ni-BCZY712/ BCZY712	300	2	microtubular	97	0.4	16
	400			97	0.96	
Ni-LMCO/LMCO/Ni-LMCO	500	1.5	planar	76	3.5	14
BZY-LSV@CeO_2-Pd/BZCYC/BZY-LSV@CeO_2-Pd	600	0.3	planar	98	2	7
Ni-BZCYYb/ BZCYYb/ Ni-BZCYYb	500	0.3	planar	98	3	8

BCZY712: $BaCe_{0.7}Zr_{0.1}Y_{0.2}O_{3-\delta}$; LMCO: $La_{1.85}Mg_{0.15}Ce_2O_{7-\delta}$; BZY: $BaZr_{0.75}Y_{0.15}O_{3-\delta}$, LSV: $La_{0.7}Sr_{0.3}V_{0.9}O_{3-\delta}$, BZCYC: $BaZr_{0.30}Ce_{0.54}Y_{0.15}Cu_{0.01}O_{3-\delta}$; BZCYYb: Ba $Zr_{0.1}Ce_{0.7}Y_{0.1}Yb_{0.1}O_{3-\delta}$.

Table S3

Ohmic Resistance and Polarization Resistance of Multilayer Hydrogen Pump Cells with Different Ba Excess Levels.

Parameter	Ohmic resistance (R_s / Ω)	Polarization resistance (R_p/Ω)	Absolute Error (R_s)	Relative Error (%) (R_s)
X=1.05	3	8.3	0.025358	0.83717
X=1.10	3.3	8.9	0.028608	0.85704
X=1.15	10.8	9.1	0.2464	2.2857
